# Hereditary Cancer Syndrome in a Family with Double Mutation in *BRIP1* and *MUTYH* Genes

**DOI:** 10.3390/genes14020428

**Published:** 2023-02-08

**Authors:** Giovanna D’Elia, Gemma Caliendo, Luana Passariello, Luisa Albanese, Jasmine Makker, Anna Maria Molinari, Maria Teresa Vietri

**Affiliations:** 1 Unity of Clinical and Molecular Pathology, AOU University of Campania “Luigi Vanvitelli”, 80138 Naples, Italy; 2 Department of GKT, School of Medical Education, King’s College London, London WC2R 2LS, UK; 3 Department of Precision Medicine, University of Campania “Luigi Vanvitelli”, 80138 Naples, Italy

**Keywords:** hereditary cancer syndrome, oncogenetic counselling, MUTYH gene, BRIP1 gene, next-generation sequencing

## Abstract

Hereditary cancer syndromes predispose to several types of cancer due to inherited pathogenic variants in susceptibility genes. We describe the case of a 57-year-old woman, diagnosed with breast cancer, and her family. The proband belongs to a family with a suspected tumor syndrome, due to other cancer cases in her family from the paternal and maternal sides. After oncogenetic counseling, she was subjected to mutational analysis with an NGS panel analyzing 27 genes. The genetic analysis showed two monoallelic mutations in low penetrance genes, c.1187G>A (p.G396D) in *MUTYH* and c.55dup (p.Tyr19Leufs*2) in *BRIP1*. One of the mutations was inherited from the maternal side and the other from the paternal side, suggesting two different cancer syndrome types in the family. *MUTYH* mutation was related to the onset of cancers on the paternal side, as confirmed by the occurrence of the same mutation in the proband’s cousin. *BRIP1* mutation was found in the proband’s mother, indicating that it was related to the cancer cases observed on the maternal side, including breast cancer and sarcoma. Advances in NGS technologies have allowed the identification of mutations in families with hereditary cancers in genes other than those related to a specific suspected syndrome. A complete oncogenetic counseling, together with molecular tests that enable a simultaneous analysis of multiple genes, is essential for the identification of a correct tumor syndrome and for clinical decision-making in a patient and his/her family. The detection of mutations in multiple susceptibility genes allows the initiation of early risk-reducing measures for identified mutation carriers among family members and to include them in a proper surveillance program for specific syndromes. Moreover, it may enable an adapted treatment for the affected patient, permitting personalized therapeutic options.

## 1. Introduction

Hereditary cancer syndromes predispose to several types of cancer due to inherited pathogenic variants in susceptibility genes. The most common hereditary cancer syndrome, autosomal dominantly inherited, includehereditary breast and ovarian cancer syndrome (HBOC), Lynch syndrome (LS), Li-Fraumeni syndrome (LFS), Cowden syndrome (CS), Peutz-Jeghers syndrome (PJS), Hereditary Diffuse Gastric Cancer (HDGC), and Familial adenomatous polyposis (FAP); whereas MUTYH-associated polyposis (MAP) presents an autosomal recessive type of inheritance [1]. Rarely, the coexistence of two different syndromes is observed in a family [2,3,4].

We observed, for the first time, a patient with double mutations in *MUTYH* and *BRIP1* genes, suggesting two different hereditary cancer syndromes in her family.

*MUTYH*, a base excision repair enzyme involved in correcting DNA errors by guanine oxidation can be considered a cellular protective factor [5]. Biallelic mutations in the *MUTYH* gene are implied in the development of MAP and were reported in 10–30% of cases [6]. Phenotypically, MAP occurs as an attenuated FAP, with less than 100 adenomas, a mean age of about 45 years at diagnosis, and an increased lifetime risk for gastrointestinal cancers. Some patients affected with MAP present with the development of serrated polyps and extracolonic manifestations [7]. Monoallelic *MUTYH* mutations associated with an extended risk of developing colorectal cancer (CC) and familial gastrointestinal diseases without polyposis have been described [8]. In the last few years, several studies investigating the impact of germline monoallelic *MUTYH* pathogenic variants (PVs) in tumorigenesis showed an increased risk for gastric, endometrial, liver, breast, ovarian, pancreatic, bladder, and duodenal cancers, as well as the onset of benign and malignant endocrine tumors [9].

*BRIP1* is involved in the maintenance of genomic integrity and acts as a tumor suppressor through its interaction with BRCA1 [10]. Mutations in *BRIP1* have been described in HBOC syndrome that predispose mostly to breast cancer (BC) and ovarian cancers (OC), but also other cancer types, including melanoma, prostatic, pancreatic, laryngeal, colorectal, and endometrial cancer [11].

Mainly, HBOC syndrome results from germline mutations in *BRCA1* or *BRCA2* genes, or other low penetrance genes, such as *PALB2*, *RAD51C*, *RAD51D*, and other DNA damage repair genes [12,13]. Several truncating variants of *BRIP1* were shown to be related to BC and OC development in HBOC syndrome [14].

Recently, a *BRIP1* mutation was found in LFS [15]. LFS is characterized by the early onset of multiple tumors, such as soft-tissue sarcomas, osteosarcomas, breast cancer, brain tumors, and leukemia. The development of cancer at age 30 has an incidence of about 50%, while at age 70, it is almost 100% [16]. About 70–80% of LFS families are carriers of germline mutations in the *TP53* gene [17,18]; however, pathogenic variants of *TP53* do not explain all cases of LFS. Mutations in the cell cycle checkpoint gene (*CHEK2*) and protection of telomere gene 1 (*POT1*) have also been described in some families without *TP53* mutations [19,20], besides *BRIP1* mutations [15].

## 2. Case Report

This study was carried out in accordance with the World Medical Association Helsinki Declaration (1964). The study was approved and conducted according to the ethical guidelines at the University of Campania “Luigi Vanvitelli” (n. 469-23 July 2019).

We describe the case of a 57-year-old woman with BC and her family. After oncogenetic counseling, the proband, belonging to a family with a suspected tumor syndrome, was subjected to mutational analysis which identified double mutations in *MUTYH* and *BRIP1* genes.

The patient was affected with left breast BC at 53 years and underwent quadrantectomy surgical treatment at the time of diagnosis. The biopsy piece showed infiltrating ductal carcinoma. The results of immunohistochemical analysis for expression levels were positive for the estrogen receptor (ER 60%), the progesterone receptor (PgR 60%), the nuclear protein Ki67 (45%), and Her2 (3+). The patient received 4 cycles of chemotherapy, Epirubicin-Cyclophosphamide with Docetaxel-Herceptin for 6 months. After this first-line therapy, she continued with Herceptin alone with Letrozolo for a period of 10 months. At the end of the treatment, mammography, ultrasonography, and magnetic resonance imaging of the breast were performed and no structural alterations were found.

**Figure 1:**
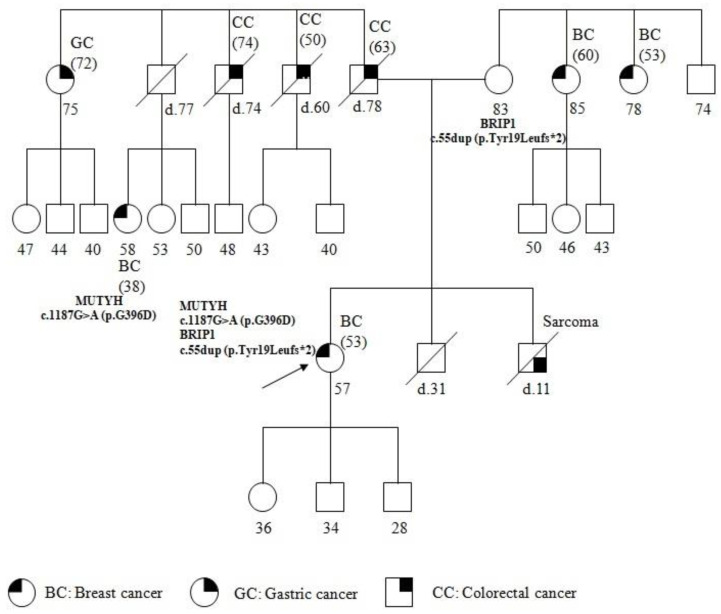
Pedigree of family carrying the *MUTYH* mutation c.1187G>A (p.G396D) and *BRIP1* mutation c.55dup (p.Tyr19Leufs*2). The age at diagnosis is indicated in brackets.

The patient underwent genetic counseling; a pedigree was generated, the personal and family histories were collected, and informed consent was signed. The pedigree of the patient and the past tumors are summarized in Figure 1. The patient reported other cancer cases in the family, on both the maternal and paternal sides. Particularly, her brother was affected with sarcoma at 11 years of age and died a few months after the diagnosis. The father, who died at 78 years, was affected with CC, diagnosed at 63 years. Moreover, two paternal uncles, both dead, were affected by CC developed at 60 and 74 years respectively. In addition, a paternal aunt developed gastric cancer (GC) at 72 years, and a paternal cousin, 38 years old, was affected with BC diagnosed at 38 years. On the maternal side, two uncles, 78 and 85 years of age, were affected with BC diagnosed at 53 and 60 years, respectively.

The pedigree analysis led to the suspected diagnosis of a cancer syndrome due to the presence of several cancers and the patient was referred formutational analysis with a cancer panel including related susceptibility genes. 

A peripheral blood sample was collected from the patient. The Wizard Genomic DNA purification kit (Promega, Fitchburg, WI, USA) was used for extraction of genomic DNA, according to the manufacturer’s instructions. Twenty ng of genomic DNA was processed with the The Hereditary Cancer Solution V1.1 (HCS) kit (Sophia Genetics, Saint-Sulpice, Switzerland). The capture based target enrichment of 27 genes including *BRCA1* (NM_007295), *BRCA2* (NM_000059), *ATM* (NM_000051.4), *CHEK2* (NM_007194), *PALB2* (NM_024675), *RAD51C* (NM_058216), *RAD50* (NM_002878), *BRP1* (NM_001003694.2), *PTEN* (NM_000314.8), *NBN* (NM_002485), *MRE11A* (NM_005591.4), *BARD1* (NM_000465.4), *STK11* (NM_000455.5), *CDH1* (NM_004360), *MUTYH* (NM_001128425.1), *TP53* (NM 000546), *MLH1* (NM_000249.4), *MSH2* (NM_000251.3), *ABRAXAS1* (NM_139076.2), *APC* (NM_000038.6), *EPCAM* (NM_002354.3), *MSH6* (NM_000179.3), *PIK3CA* (NM_006218.4), *PMS2* (NM_000535.7), *PMS2CL* (NM_000535.7), *RAD51D* (NM_002878.4), and *XRCC2* (NM_005431.2), and the library construction protocols were carried out according to the procedure described by the manufacturer.

Library quantification was effected with fluorometric quantitation employing the Qubit dsDNA High Sensitivity kit (Thermofisher Scientific, Waltham, MA, USA). As quality control, the profile of each sample obtained was analyzed, using Bioanalyzer DNA 1000 (Agilent Technologies, Santa Clara, CA, USA).

Sequencing was performed on Illumina MiSeq (Illumina, San Diego, CA, USA) platform, as described by SOPHiA Genetics’ protocols, and sequencing was obtained on a 600-cycle format V3 flow-cell. Sequencing data were elaborated for single nucleotide variants (SNVs), and copy number variations (CNVs) via the SOPHiA DDM platform based on SOPHiA Artificial Intelligence (AI).

The regions of interest (ROIs) were defined as exons ±50 base pairs of intronic sequence for all genes. Target regions showed an average read coverage of 900× with a minimum depth of >50× for 99% of bases. Variants were called with a variant allele frequency (VAF) cut-off of 20%.

Mutational analysis was also carried out for the mother and the paternal cousin of the patient.

Sanger sequencing on the other blood sample was used to confirm the presence of a point mutation, as described [21,22,23]. Molecular analysis in the family members of the probands with mutation was performed by Sanger sequencing [21,22,23]. The results were elaborated using Mutation Surveyor^®^ software, version 3.24 (Softgenetics, State College, PA, USA).

For the identification and classification of genetic variants, ClinVar and LOVD databases were used. Genetic variants found were categorized according to criteria by International Agency for Research on Cancer recommendations [24] and categorized in classes as benign (class I), likely benign (class II), variant of uncertain significance (VUS, class III), likely pathogenic (class IV), and pathogenic variants (PVs, class V).

**Figure 2:**
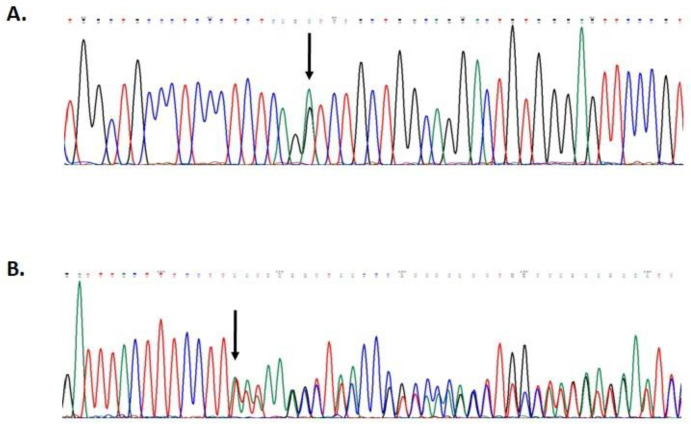
Partial electropherogram of *MUTYH* exon 13 (**A**); partial electropherogram of *BRIP1* exon 1 (**B**).

**Figure 3:**
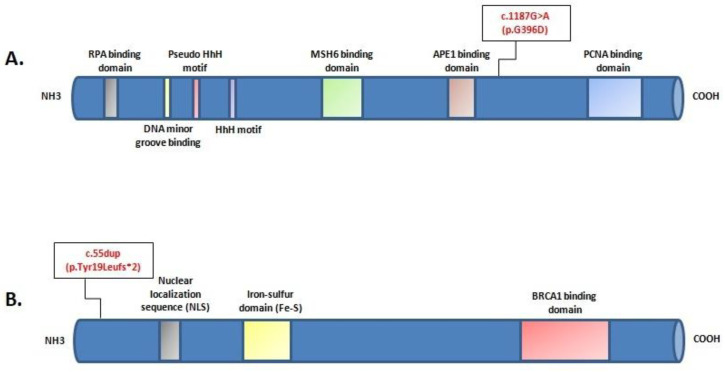
Locationof c.1187G>A (p.G396D) mutation in the protein structure of MUTYH (**A**); location of c.55dup (p.Tyr19Leufs*2) mutation in the protein structure of BRIP1 (**B**).The position of each mutation is shown with respect to domains of functional significance in the translated protein.

Mutational screening revealed two monoallelic mutations in *BRIP1* and *MUTYH* genes (Figure 2). The *MUTYH* mutation, c.1187G>A (p.G396D), is localized in exon 13 of the gene and is classified as pathogenic, class V. The *BRIP1* mutation, c.55dup (p.Tyr19Leufs*2), is localized in exon 1 of the gene (Figure 3). This variant is also classified as pathogenic, class V.

The genetic analysis showed the same *BRIP1* mutation in the healthy mother of 83 years, and the same *MUTYH* mutation in the paternal cousin with BC. It has not yet been possible to extend the molecular analysis to the patient’s children and other family members.

## 3. Discussion

We report the case of a patient affected with BC. The mutational analysis showed two monoallelic PVs, in *MUTYH* gene and *BRIP1* gene, one inherited from the maternal side and the other from the paternal side, suggesting the presence of two different cancer syndrome types in the family. Indeed, onthe paternal side, cases of CC and GC occurred, consistent with familial gastrointestinal diseases, and a BC casewas found, all of which are associated with *MUTYH* monoallelic mutations [9].

The c.1187G>A (p.G396D) biallelic mutation is most commonly found in MAP patients [25] and is localized to a highly conserved amino acid region in *MUTYH*. It has been determined to reduce the capacity of binding the substrate and to impair glycosylase activity [26]. It is also the most common monoallelic mutation and is found in cases of colorectal, gastric, breast, and lung cancers [8]. An increased risk of BC was found in women with MAP; moreover, heterozygous *MUTYH* mutations, including p.Tyr179Cys, p.Gly396Asp, and p.Pro405Leu were found infamilies with both BC and colorectal cancer [9]. In the proband’s family, *MUTYH* PV is related to the onset of cancers on the paternal side, as confirmed by the occurrence of the same *MUTYH* PV in the proband’s cousin, who was affected with BC (Figure 1).

On the proband’s maternal side, two BC cases occurred, suggesting HBOC syndrome. However, the presence of a case of sarcoma, in the proband’s brother, who died at 11 years of age, directs to LFS, where cases of both BC and sarcoma arise [16]. Mutations in *BRIP1* have been described to be associated with cases of breast as well as ovarian cancers [14], and, recently, with LFS [15].

Thus, the occurrence of double mutations in *MUTYH* and *BRIP1* genes in the patient was a rare and random event and the mutations were inherited from the maternal and paternal sides, respectively. To date, no double heterozygosity in the *MUTYH* and *BRIP1* genes has been described.

In many cases, due to the high frequency of recurrent tumors, some families satisfy the genetic testing criteria for more than one hereditary syndrome, thus allowing the identification of more than one inherited syndrome. Before the implementation of next-generation sequencing (NGS) in clinical diagnostics, the individuation of a germline mutation had been limited to patients who met the clinical criteria of a specific syndrome, where only genes related to that syndrome were analyzed. A negative result could implicate a mutation in genes that were not analyzed. Advances in NGS technologies have allowed the identification of PVs in families with hereditary cancers in genes other than those related to a specific suspected syndrome. NGS allows the simultaneous evaluation of multiple cancer-predisposing genes, using panels including up to 500 cancer-related genes, and thus improve the identification of disease-associated variants in low penetrance genes [27,28,29].

In our proband, the oncogenetic counseling highlighted the suspicion of more than one probable cancer syndrome in the family. The results of the mutational analysis with a sequencing panel including many cancer-associated genes allowed the detection of two mutations in low penetrance genes and confirmed the presence of two syndromes.

A complete oncogenetic counseling in addition to accurate mutational analysis is essential for diagnosis and clinical decision-making in a patient and his/her family. For instance, in this case, the patient met the criteria for HBOC, LS, as well as LFS, but if the sequencing panel did not include many cancer-associated genes, we could not have detected PVs in low penetrance genes.

In hereditary cancer, the use of molecular tests that allow a simultaneous analysis of multiple genes, enables us to identify a complex genotype that contributes to specific phenotypic conditions. Moreover, the individuation of a specific syndrome allows a proper surveillance program [30,31]. In our case, the mutational analysis of multiple cancer-related genes enabled us to identify mutations in loss-risk genes and to include the proband and her mutated family members in a specific surveillance program for related cancers onset.

To date, the proband does not report any gastrointestinal diseases, she is enrolled in a surveillance program involving biennial colonoscopy, as established by guidelines due to the risk associated with the *MUTYH* PVs [32]. In addition, the patient is also monitored for contralateral BC and OC, due to the risk associated with *BRIP1* mutation, as indicated by NCCN guidelines [33]. Likewise, the mother, carrying the same *BRIP1* mutation, although healthy, is also enrolled in the surveillance program. In the future, the proband’s children will also undergo genetic testing to evaluate the inheritance of the mutations.

Therefore, a complete oncogenetic counseling, in addition to molecular tests that enable a simultaneous analysis of multiple genes, can improve the clinical management of cancer families, as this allows appropriate risk management for confirmed mutation carriers among family members. In addition, it offers the potential for establishing targeted follow-up protocols for related cancers onset, and personalized therapeutic options for the affected patient.

## 4. Conclusions

An effective clinical management can be actuated in the families when a genetic predisposition has been identified. Traditionally, genetic screening analyzed the classical high penetrance genes that explained the genetic predisposition only in a few cases. With development of gene cancer panels, which not only include high-penetrance susceptibility genes but also genes with lower penetrance, using NGS-based technology, it is possible to explore several genes at once, increasing the chance of finding a causal mutation or a double mutation in two genes.

A critical decision regarding the multigene panel testing is to look into the right genes for the particular hereditary syndrome. In some families, the cancer cases can meet the criteria for different syndromes, due to the overlapping phenotypes, therefore, the panel should include the candidate genes that match with the phenotype of the patients.

Therefore, genetic counseling with a careful analysis of the pedigree is essential in the identification of hereditary cancer syndromes occurring in families. Indeed, the detection of mutations in multiple susceptibility genes is critical, as it allows the initiation of early risk-reducing measures for confirmed mutation carriers in the family. In addition, it may enable an appropriate treatment strategy for the patient.

In the last few decades, the increasing characterization of cancer syndromes through gene profiling has allowed a greater understanding of the molecular mechanisms underlying cancer, thus permitting better, more personalized therapeutic options. The use of Poly (ADP-ribose) polymerase (PARP) inhibitors has shown positive results in patients with *BRCA* mutations and changed the standard of care in several cancers including HBOC-syndrome-associated breast, ovarian, pancreatic, and prostatic cancers [34,35].

In our case, the use of a multigenic panel allowed the detection of mutations in low penetrance genes or in genes not directly related to the initial established phenotype according to family history, that would have gone unnoticed with traditional analysis.

## Data Availability

Not applicable.
